# Genetics and Epigenetics of Atrial Fibrillation

**DOI:** 10.3390/ijms21165717

**Published:** 2020-08-10

**Authors:** Estefanía Lozano-Velasco, Diego Franco, Amelia Aranega, Houria Daimi

**Affiliations:** 1 Department of Experimental Biology, University of Jaen, 23071 Jaen, Spain; evelasco@ujaen.es (E.L.-V.); dfranco@ujaen.es (D.F.); aaranega@ujaen.es (A.A.); 2 Medina Foundation, Technology Park of Health Sciences, Av. del Conocimiento, 34, 18016 Granada, Spain; 3 Biochemistry and Molecular Biology Laboratory, Faculty of Pharmacy, University of Monastir, Monastir 5000, Tunisia

**Keywords:** atrial fibrillation, gene regulation, epigenetics

## Abstract

Atrial fibrillation (AF) is known to be the most common supraventricular arrhythmia affecting up to 1% of the general population. Its prevalence exponentially increases with age and could reach up to 8% in the elderly population. The management of AF is a complex issue that is addressed by extensive ongoing basic and clinical research. AF centers around different types of disturbances, including ion channel dysfunction, Ca^2+^-handling abnormalities, and structural remodeling. Genome-wide association studies (GWAS) have uncovered over 100 genetic loci associated with AF. Most of these loci point to ion channels, distinct cardiac-enriched transcription factors, as well as to other regulatory genes. Recently, the discovery of post-transcriptional regulatory mechanisms, involving non-coding RNAs (especially microRNAs), DNA methylation, and histone modification, has allowed to decipher how a normal heart develops and which modifications are involved in reshaping the processes leading to arrhythmias. This review aims to provide a current state of the field regarding the identification and functional characterization of AF-related epigenetic regulatory networks

## 1. Introduction

Atrial fibrillation (AF) is known to be the most common supraventricular arrhythmia affecting up to 1% of the general population [1,2]. Its prevalence exponentially increases with age and could reach up to 8% of the elderly population (age > 80 years), representing thus one of the most significant global health burdens [3]. The presence of AF substantially contributes to morbidity and mortality by significantly affecting patient’s quality of life and increasing the risk of embolic stroke and heart failure. Beyond age, there are many types of cardiac and medical conditions that confer increased AF risk. These include arterial hypertension, cardiomyopathies, obstructive sleep apnea, or valvular dysfunction [4,5]. For years, AF has not been considered a genetic condition. However, the discovery of the first lone AF in a family with an autosomal dominant pattern of inheritance gave evidence of a genetic contribution in the development of AF [6]. Subsequently, several studies have further demonstrated that AF and in particular, lone AF, have a substantial genetic basis [7,8,9,10]. Familial studies along with population-based genome-wide association studies (GWAS) have shed light on genetic mutations and polymorphisms, which associate with AF and explain in part its heritability [11]. A recent meta-analysis of GWAS for AF identified over 100 AF risk loci [12]. However, identification of risk loci is only the start of a long process to discover the mechanisms by which these variants increase AF risk.

Genetic variants can contribute to the AF pathophysiology by altering structure and therefore, the expression and function of proteins responsible for various cellular activities [13]. It is recognized that heterogeneity in gene expression among cells and individuals is in part due to an interaction of a plethora of environmental factors and individual lifestyles but the link between these external risk factors and the internal genetic machineries has been unclear. The discovery of epigenetics has helped the scientific community to explain the link between genes and the environment. The term “epigenetics” is defined as changes in gene expression that cannot be explained by changes in DNA sequence [14] but rather result from alterations related to the packaging and/or translation of genetic information [15]. Multiple diverse epigenetic processes, including the expression of non-coding RNA molecules, DNA methylation and histone modification influence the expression of genes which in turn lead to drastic changes in the cellular structure and function, influencing thus the organism response to diseases [16]. Epigenetic mechanisms can be acquired or inherited and constitute a mean by which the cardiovascular transcriptome is controlled in a well-coordinated manner during development and disease. Although, the role of epigenetic mechanisms in cardiovascular diseases (CVDs) has been under intense scrutiny during the last few years [17], its role in AF onset and development is still poorly elucidated. AF is starting to be viewed as much more complex and dynamic disorder as more epigenetic mechanisms are discovered. Thus, important insights are expected to emerge in the near future, including novel AF biomarkers and new therapeutic targets. In the course of this review, we will discuss the cellular and molecular basis of AF as well as the contribution of epigenetics in the disease onset and development.

## 2. Clinical and Pathophysiologic Basis of Atrial Fibrillation

### 2.1. Classification

Atrial fibrillation (AF) is the subject of several overlapping schemes of classification in which the subgroups are often poorly defined. As any disease, AF could be symptomatic or silent depending on the manifestation of its related complications [18]. However, the most used classification is according to its temporal pattern. AF is generally considered as paroxysmal AF when the fibrillatory episodes self-terminate within seven days. Paroxysmal AF may progress to persistent and finally chronic or permanent states that fail to self-terminate [19]. The disease aetiology is often used as a classification criterion as well. AF is described as lone AF when it occurs without evidence of other cardiac or systemic disease known to promote AF. Thus, it is no longer considered as lone when it concurs with hypertensive, valvular, or other structural heart diseases [20].

### 2.2. Pathophysiology of AF

In health, differences in action potential duration and refractory period between the ventricular and atrial myocardium make this later more prone to the development of very rapid rates with complex patterns of conduction than the ventricular myocardium [21]. In the atrial fibrillation condition, these physiological differences favorize the rapid and disorganized atrial activation which leads to impaired atrial function [22]. Although decades of investigation on the triggers and the sustainer factors of the disease, AF mechanisms remain incompletely understood and thus the disease poorly treated [23]. However, a commonly accepted mechanism of AF assumes that the lost synchronization of atrial contraction, results from randomly propagating waves with intermittent blockades, annihilation, and re-generation of discrete waves. This rapid and uncoordinated atrial activity can be diagnosed on an EKG by lack of a P-wave and irregular QRS complexes [24].

Current research on AF support the fact that initiation and maintenance of AF require pathophysiological remodeling of the atria, either specifically as in lone AF or secondary to other heart disease as in heart failure-associated AF [25].

### 2.3. Electrical and Structural Remodeling in AF

Atrial remodeling refers to any persistent change in atrial structure or function [26]. This remodeling could be caused by the AF itself leading to electrophysiological, contractile, and structural changes [27,28,29]. Atrial remodeling most likely starts with electrical remodeling characterised by changes in atrial refractoriness and slowed conduction time. These changes occur due to alterations in action potential currents, especially Ca^2+^ influx and its subsequent homeostasis [30]. The rapid atrial pace rises the Ca^2+^ inward currents, increasing thus the internal cellular Ca^2+^ load with each action potential. This triggers the autoprotective cellular mechanisms that reduce Ca^2+^ entry. Reduced L-type Ca^2+^ (𝐼_CaL_) current, Ca^2+^ overload, changes in K^+^ current (𝐼_KACh_, 𝐼_K1_), Na^+^ current (𝐼_Na_), and transient outward current (𝐼_to_) have each been reported in AF [31]. All these changes stabilize atrial re-entry rotors, increasing AF vulnerability and sustainability [32,33]. In addition, alterations in Ca^2+^ handling promote ectopic activity and diastolic Ca^2+^ leakage from the sarcoplasmic reticulum (SR) into the cytoplasm via ryanodine receptors (RyRs) [34]. In AF, RyRs are also subject to remodelling, via RyRs phosphorylation, in a manner that increases calcium release [35,36]. Electric remodeling thus favours the development of functional re-entry substrates, which are reversible on AF termination (reverse remodeling) and contribute to persistent AF. As atrial disease progresses to irreversible structural changes, AF becomes permanent [27].

Atrial structural remodeling includes inflammation, cell hypertrophy, atrial dilation, and fibrosis, which cumulatively contribute to abnormal electrical signal formation and conduction as an arrhythmogenic substrate [37,38]. In fact, the increased atrial wall stress (stretch) and decreased cardiac output resulting from the lack of synchronization between the rapid atrial activity and the irregular ventricular response are suggested to be beyond the elevated hemodynamic load during AF [39]. Altered atrial hemodynamic load enhances the release of angiotensin II, by activating the renin–angiotensin–aldosterone system, and is associated with endothelial damage and the recruitment of cytokine-secreting inflammatory cells [40]. Furthermore, the elevated hemodynamic load on the atria promotes cellular hypertrophy, cardiomyocyte dysfunction, myocyte death (through apoptosis and necrosis), and fibrosis [41]. Among all, atrial fibrosis is the major characteristic of AF-related remodeling [42]. Atrial fibrosis is characterized by an excessive fibroblast proliferation and accumulation of collagenous material in the extracellular space resulting from an imbalance between extracellular matrix (ECM) deposition and degradation within the heart [43]. Atrial fibrosis is also proposed to alter both total gap junction proteins expression and distribution along cell membrane creating a disruption, most likely decrease, in cellular communication between cardiomyocytes [9].

## 3. Genetics of Atrial Fibrillation

A number of recent studies have demonstrated that AF and in particular, lone AF, has a substantial genetic basis [7,8]. Although factors such as sex, ageing, and comorbidities contribute to AF risk, different studies have shown that a family history of AF confers an increased risk to the disease [8,44,45]. In fact, it has been reported that having a parent with AF approximately doubled the four-year risk of developing AF, even after adjustment for AF risk factors previously cited [45]. Chen and colleagues identified the first AF mutation responsible for an autosomal dominant form of familial AF [46]. The identified mutation, namely the S140G, is localized in KCNQ1 gene that encodes for the pore forming α-subunit of the Kv7.1 voltage-gated potassium channel responsible for the slow component of the delayed rectifier potassium current (I_Ks_). Upon demonstrating the heritable component of AF, several studies have emerged to identify the genetic basis of the disease. Most of the early studies employed the common research strategy which consists of selecting the genes of interest using previous knowledge of gene function, typically referred as “candidate gene study”.

**Figure 1:**
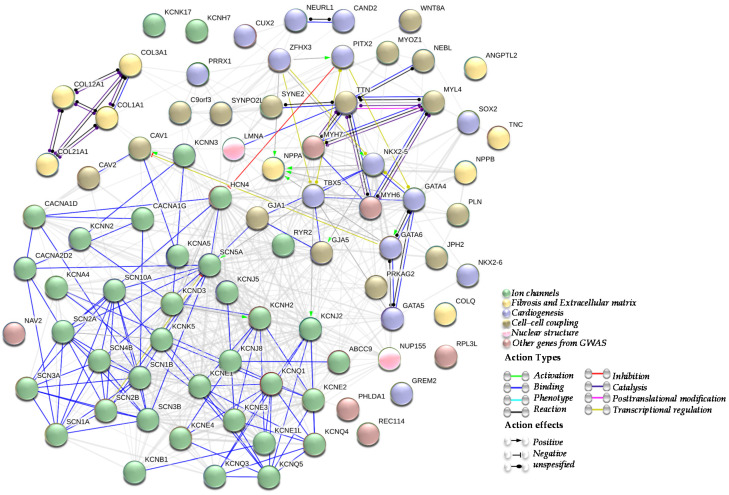
Atrial Fibrillation (AF) genes network. A network summarizing the interactions between genes described in association with atrial fibrillation. Being an arrhythmogenic disorder in a first place, most of genes associated with AF are encoding for cardiac ion channels (green cluster). Other AF genes are implicated in fibrosis and extracellular matrix (ECM) structure (yellow cluster), cardiogenesis (blue cluster), cell-cell coupling (brown cluster) and nuclear structure (pink cluster). Other AF genes identified by genome-wide association studies (GWAS) are represented as well (dusty pink cluster). All those clusters are in line with the electrical and structural remodelling that represent the AF substrate.

Subsequently, several studies identifying numerous other AF candidate genes and risk loci, such as ion channels, genes involved in fibrosis and the extracellular matrix (ECM) remodelling, genes involved in cardiogenesis, genes implicated in the cell-cell coupling or nuclear structure, and genes and/or loci identified by GWAS, have emerged (Figure 1).

### 3.1. Ion Channel Genes in AF

The cardiac action potential (AP) reflects the electrical activity of the cardiomyocytes during the contraction and relaxation of the heart and it is initiated and maintained by specific ion currents contributing to each phase of it. An AP is initiated by an upstroke phase occurring due to rapid transient influx of Na^+^ (I_Na_) that is ensured by Nav1.5 sodium channels. Later, Na^+^ channels are inactivated, combined with a transient efflux of K^+^ (I_to 1,2_). The second phase of AP, also known as the plateau phase, is ensured by a counterbalance of K^+^ efflux (I_Ks_) and Ca^2+^ influx (I_ca-L_). The sustained repolarization (phase 3) at the end of the plateau is governed by the predominance of K^+^ efflux, via the slow (I_Ks_) and the rapid (I_Kr_) delayed rectifier as well as the inward (I_K1_) rectifier potassium currents. Finally, as part of phase 4, resting potential in myocytes is maintained by series of cardiac potassium channels.

Given the initial identification of the S140G mutation in KCNQ1, [46] many investigators then explored ion channel genes involved in the cardiac action potential, and a cascade of mutations have subsequently been identified (Table 1).

#### 3.1.1. Potassium Channels Mutations

Unlike the KCNQ1 (S140G) mutation which resulted in a gain of function of I_Ks_ channels, Otway and colleagues identified a missense mutation (R14C) in the same gene that had no significant effect on I_Ks_ current amplitudes in cultured cells at baseline [47]. However, among the R14C mutation carriers, only those with left atrial dilatation had AF. Similarly, gain-of-function mutations identified in KCNQ1 channel were shown to induce altered I_Ks_ activity and kinetics, thereby increasing the arrhythmogenicity to AF, most likely by shortening atrial but not ventricular action potential [48,49,50,51,52,53]. However, loss-of-function mutations in the I_Ks_ channel have previously been described as well in familial AF suggesting thus that both gain-of-function and loss-of-function of cardiac potassium currents enhance the susceptibility to AF [54]. 

Several studies linked the heterogeneity of KCNQ1 mutation type (either gain-of-function or loss-of-function) in AF to the structural relationship between KCNQ1 and its β-subunits in the I(_Ks_) channel [55,56]. Lundby et al. [55] demonstrated that the Q147R mutation in KCNQ1 represents a ‘loss-of-function’ KCNQ1 mutation (molecular substrate for AF) when co-expressed with KCNE1, but a ‘gain-of-function’ mutation when co-expressed with KCNE2, alone or with KCNE1 (molecular substrate for QT prolongation). Thus, it is no surprise that mutations in KCNE1, KCNE2, KCNE3, KCNE4 and KCNE5, encoding regulatory β-subunits of Kv7.1, have been identified in AF and been demonstrated to exert gain-of-function effects on I_Ks_ and to have potential effect on I_to_ and I_Kr_ [55,57,58,59,60,61,62,63]. Although KCNE1-5 and KCNJ2 mutation have been demonstrated by Ellinor et al. [64] to rarely cause typical atrial fibrillation in a referral clinic population, impairment of the KCNJ2, KCNJ5, and KCNJ8 channels, responsible for the resting membrane potential, has been demonstrated to promote AF initiation [65,66,67,68]. Further evidence supporting the role of potassium channels in AF comes from the identification of loss-of-function mutations in the *KCNA5* gene, encoding for the Kv1.5 voltage-gated potassium channel responsible for the ultra-rapid component of the delayed rectifier potassium current, in families with hereditary lone AF [69,70]. Furthermore, it has been demonstrated that around 30% of short QT syndrome patients who carry KCNQ1 or KCNH2 gain-of-function mutations, have frequent paroxysms of AF [49,71]. Gain-of-function mutations in KCND3 have previously been described in early-onset lone AF. This association of K_V_4.3 gain-of-function and early-onset lone AF further supports the hypothesis that increased potassium current enhances AF susceptibility [72]. However, downregulation of other sodium channels such as SK2 (encoded by KCNN2 gene) and SK3 (encoded by KCNN3 gene) was observed in human chronic AF as well [73] and ablation of SK2 channels resulted in a delay in cardiac repolarization and atrial arrhythmias [74]. Loss-of-function missense mutation was also identified in the ABCC9, a gene encoding the SUR2A K_ATP_ channel subunit involved in maintaining electrical stability under stress, in a female case with early-onset AF [74]. 

Mutations in potassium/sodium channels have been also identified in AF. Thus, HCN4 mutations have also been reported in association with AF [74]. HCN4 is expressed in the sinoatrial (SA) node and underlies the I_f_ current normally responsible for the pacemaker current in nodal myocytes. Mutations of this channel may lead to a diminished action potential frequency (heart rate slowing) and delays after depolarizations that might trigger AF [75].

#### 3.1.2. Sodium Channels Mutations

Mutations in SCN5A, the gene encoding for the α-subunit of the cardiac sodium channel responsible for the I_Na_ current, were long associated to ventricular fibrillation syndromes such as Long QT syndrome type 3 (LQT3) and Brugada Syndrome (BrS). However, Olson and colleagues identified the first SCN5A missense mutation that was associated with several cardiac phenotypes including AF [76]. Subsequently, multiple mutations in SCN5A gene have been identified in patients with AF, alone [77,78,79,80,81] or with combined cardiac conditions [76,82,83,84,85,86]. Several variants of SCNA5 have been reported in patients with common polygenic AF as well [87]. Recently, Olesen et al. [88] identified eight mutations in SCN5A in a cohort of lone AF patients. These mutations presented overlapping functional effects: compromised transient peak current and increased sustained current indicating that both gain- or loss-of-function alterations in cardiac sodium current constitute a substrate of early-onset AF. 

Mutations in the four β-subunits SCN1-4B have been reported to attenuate sodium currents and shift the voltage-dependence of sodium channel gating and to be associated to AF [89,90,91,92,93]. Furthermore, a genetic variant in SCN10A, which encodes Nav1.8, a voltage gated sodium channel that participates in the late sodium current, was recently described to be associated with early onset AF [94]. Taken together, these findings suggest that impaired I_Na_ current might promote AF, yet electrophysiological evidence is only available for a small subset of these point mutations. 

#### 3.1.3. Impaired Calcium Homeostasis and AF

As explained previously, alterations in Ca^2+^ handling promote ectopic activity and diastolic Ca^2+^ leak from the sarcoplasmic reticulum (SR) into the cytoplasm via ryanodine receptors (RyRs) creating thus an electrophysiological substrate for the AF onset and development [34]. Recently, mutations in RYR2 have been associated to AF [95]. Zhang and colleagues [96] recently described an RyR2-P2328S mutant mice model presenting impaired calcium homeostasis associated to an acute atrial arrhythmogenicity while Weeke et al. [97] performed a whole exome study (WES) in families with early onset lone and they identified two disease segregating rare variants in CACNB2 (encoding the β2-subunit of the L-type calcium channel) and CACNA2D4 (encodes an l-type calcium-channel auxiliary subunit of the alpha-2/delta subunit family) with overlapping effects on the Cav1.2 current suggesting that these variants could identify an important pathway modulating AF susceptibility. Collectively, these finding bring supplementary evidences on the role of calcium homeostasis imbalances in AF pathophysiology.

### 3.2. AF Genes Involved in Fibrosis and Extracellular Matrix (ECM) Remodeling 

Mutations in genes involved in atrial structural remodelling were identified in AF patients as early as ion channel mutations (Table 1). The first non-ion channel AF mutation was an NPPA frameshift variant reported by Hodgson-Zingman et al. [98]. Subsequently, several other variants in NPPA were later also linked to AF [99,100]. Cheng et al. [101] suggested that NPPA variants promote AF by activating inflammation and fibrosis. However, the exact molecular mechanism by which NPPA variants cause AF is poorly understood.

Thomas and colleagues [102] have recently identified a cascade of genes differentially expressed in atrial samples from AF patients. Interestingly, samples from the right atrium showed an upregulation of genes engaged in the extracellular matrix (ECM) organization and collagen formation and collagen degradation pathways and the clathrin-coated endocytic vesicle membrane gene ontology term. Among the identified genes, MMP3 (member of the matrix metalloprotease family), COMP (cartilage oligomeric matrix protein) and COL12A1/COL23A1 (collagen-encoding genes) presented an impaired expression in right atria. Other genes such as COL21A1, ANGPTL2, and COLQ were enriched in both left and right atria, giving further evidence of their role in early onset of AF by promoting atrial fibrosis and triggering structural remodeling in the atria.

### 3.3. AF Genes Involved in Cardiogenesis

Several mutations in developmental genes have been identified in AF, including genes encoding transcription factors (TFs) and growth factors involved in cardiogenesis [103] (Table 1). Particularly, loss-of-function mutations in GATA4 were identified in heterogenous AF contexts ranging from familial lone AF, to sporadic lone AF [104,105,106]. Furthermore, genetic variants in GATA5 and GATA6 genes were identified in probands with familial AF, alone or combined with congenital cardiac defects, resulting in decreased transcriptional activity [107,108,109,110]. 

It is currently well established that GATA4 and GATA6 genes work synergistically with NKX2-5, a homeobox-containing transcription factor involved in cardiac development and septation, in the regulation of target gene expression, especially those involved in cardiogenesis [111]. Thus, it is no surprise that multiple NKX2-5 mutations have been causally implicated in AF [112,113,114] Wang et al., reported the first NKX2-6 mutation in a patient with lone AF [115]. Suggesting that mutant NKX2-6 may presumably contribute to the development of AF through a similar transcriptional mechanism than NKX2-5.

Recently, gain-of-function genetic variants in GREM2, that encode the bone morphogenetic protein (BMP) antagonist gremlin-2, have been reported in probands with lone AF [116]. Giving the important role of GREM2 in cardiac laterality establishment and atrial differentiation, mutations in this gene may bring further evidences on its involvement in the early onset of AF.

### 3.4. AF Genes Implicated in the Cell-Cell Coupling

Genes involved in cell-to-cell impulse propagation have been long targeted by AF-related studies due to their crucial role maintaining cell communication and electrical coupling between atrial myocytes (Table 1). Investigators identified somatic and germline mutations in in both GJA5 (encoding Cx40) and GJA1 (encoding Cx43) in lone AF patients [117,118]. The electrophysiological characterization of connexin mutations showed either gain or loss-of-function of gap junction (GJs) channels due to an impaired trafficking or channel formation. Thus, such functional alterations may lead to AF [119,120].

### 3.5. AF Genes Implicated in Nuclear Structure

Mutations in structural genes such as NUP155 (encodes a nucleoporin) and LMNA (encodes lamin A/C) have been reported in familial AF [121,122] (Table 1). Recently, Han and colleagues reported Lamin A/C mutation that weakens the interaction between lamin A/C and NUP155, leading to the development of AF and providing a novel molecular mechanism for the pathogenesis of AF [123].

### 3.6. Other AF Genes and/or Loci Identified by GWAS

Genome-wide association studies (GWAS) are unbiased correlation studies designed to identify associations between allele frequencies and trait variation. With the advances of GWAS, the search for the genetic components of AF has relentlessly accelerated. 

The first GWAS in the AF context was performed by Gudbjartsson et al. in 2007 [124]. The study identified two SNPs, namely rs2200733 and rs10033464 localized on chromosome 4q25, conferring a high risk to AF in European and Chinese populations. Although the mechanism of action of these variants is still unclear, their genetic localization in an intergenic region, with the nearest gene, PITX2 (encodes the paired-like homeodomain transcription factor 2), 150 kilobases (kb) away, suggests a plausible role of both SNPs in the pathogenesis of AF [125,126] In fact, experimental studies in mice, including ours, demonstrated that Pitx2 is crucial for embryonic development, atria and sinus node formation/function, and left-right heart asymmetry [127,128,129]. Impaired Pitx2 function was demonstrated to increase risk of atrial arrhythmias [130]. Thus, one can speculate that these variants may alter the function of PITX2 either in early development or in adulthood and thus predispose to AF.

Since the description of the 4q25 risk loci, subsequent GWA studies emerged either to validate Gudbjartsson et al. findings in different populations [131] or reveal more AF risk loci. By the end of 2016, more than 18 SNPs (including 4q24 variants) were mapped close to 15 protein coding genes: PITX2 (4q25), ZFHX3 (16q22), KCNN3 (1q21), CAV1 (7q31), PRRX1 (1q21), C9ORF3 (9q22), HCN4 (15q24), SYNPO2L/MYOZ1 (10q22), CAND2 (3q25), GJA1 (6q22), NEURL (10q24), CUX2 (12q24), TBX5 (12q24), SYNE2 (14q23), and WNT8A (5q31) [132] (Table 2). To date, more than 100 AF risk loci are identified by GWAS [12]. Interestingly, several studies complementarily demonstrate that most of these GWAS identified genes are either directly or indirectly interconnected with PITX2 and that all of them are engaged in a big network favorizing AF’s substrate and contributing to the disease onset and progress [128,130,133,134]. Although the extensive employment of GWAS technology has generated multiple novel hypotheses of AF pathophysiology, the mechanistic role of most risk loci for AF identified using GWAS remains unknown. 

Most of the AF-associated SNPs are found in intergenic/intronic regions rather than protein-coding regions. Thus, it is thought that the different AF risk variants may act in an additive way to epigenetic factors to cause the disease. 

## 4. Epigenetics of Atrial Fibrillation

### 4.1. microRNAs and Atrial Fibrillation

**Figure 2:**
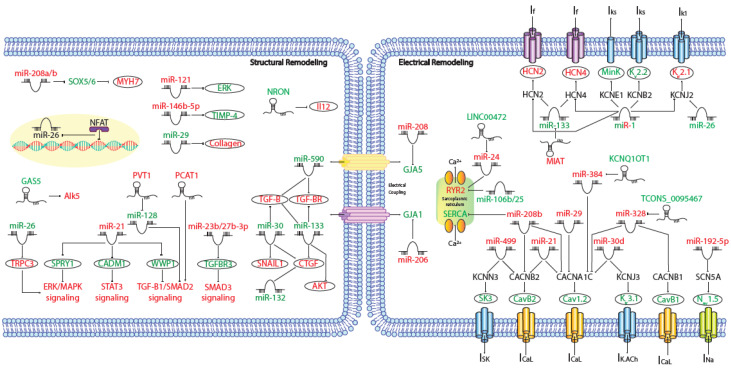
Schematic representation of the activated molecular program in atrial fibrillation. miRNAs and lncRNAs networks controlling structural and electrical remodelling in AF. (Red and green labels correspond with induced or repressed molecules in AF, respectively).

MicroRNAs (miRNAs) are small (~19–25 nt) non-coding RNAs that are encoded by nuclear DNA and transcribed by RNA polymerase II. miRNAs main function is regulating gene expression post-transcriptionally through binding to complementary target sites within mRNAs, normally within the 3′UTR. This generally results in the inhibition of translation and/or degradation of the target transcript [135,136]. At present, multiple microRNAs have been involved in electrical and structural remodeling directly linked to the course of atrial fibrillation (Figure 2) as detailed below.

#### 4.1.1. microRNAs and Electrical Disturbances in AF

I_K1_ disturbances are the most relevant ionic current changes underlying AF-induced electrical remodeling. As mentioned before, an increase in I_K1,_ is a prominent feature of AF electrical remodeling and related to this process several miRNAs are involved. MiR-1 is a muscle-specific miRNA and the most abundantly expressed miRNA in both ventricles and atria [137]. It has been shown that down-regulation of miR-1 has proarrhythmic effect modulating I_K1_ through an up-regulation of potassium inwardly rectifying channel subfamily J member 2 (*KCNJ2)* expression [138]. Concomitantly, miR-26 is significantly reduced in AF patients compared to controls, leading to an I_K1_ increase, by direct targeting of *KCNJ2*. Nuclear factor of activated T cells (NFAT), a known actor in AF-associated remodeling, was found to negatively regulate miR-26 transcription [139]. Moreover, other groups have demonstrated that an up-regulation of miR-1 accelerates the shortening of the atrial effective refractory period (AERP) by targeting potassium voltage-gated channel subfamily E regulatory subunit 1 (*KCNE1)* and potassium voltage-gated channel subfamily B member 2 (*KCNB2*) [140]. Also, miR30d is significantly upregulated in cardiomyocytes from AF patients, whereas the mRNA and protein levels of KCNJ3/Kir3.1, postulated target of miR-30d, are markedly reduced, concomitant with a reduction of the acetylcholine-sensitive inward-rectifier K^+^ current (I_K.ACh_) [141]. Furthermore, miR-499 is up-regulated in permanent AF patients, this miRNA targets and down-regulates potassium calcium-activated channel subfamily N member 3 (*KCNN3*) and facilitates its recruitment into miRISCs, resulting in transcriptional repression of the small conductance calcium-activated potassium channel protein 3 (*SK3*) expression [142] (Table 3).

Aside from I_K1_ remodeling, sodium channel (I_Na_) density may be reduced in AF. In this context, it has been stablished that an upregulation of miR-192-5p in AF patients is corresponded to downregulation of SCN5A and Nav1.5 protein [143] (Table 3).

In addition, AF is characterized by a prominent downregulation of I_CaL_ current and calcium handling remodeling. In this context, miR-21, whose expression levels are increased in myocytes isolated from chronic atrial fibrillation patients, decreases I_CaL_ by downregulating calcium voltage-gated channel subunit alpha1 C (*CACNA1C*) and calcium voltage-gated channel auxiliary subunit beta 2 (*CACNB2*) [144]. Something similar happens with miR-29 and miR-30d, targeting *CACNA1C* [141,145]. Microarray screen in AF patients identified miR-208a and miR-208b, in particular, as the most significantly increased miRNAs in AF, miR-208b over-expression analysis showed that aberrant miR-208b levels reduce the expression and function of *CACNA1C* and *CACNB2* [146]. Additionally, miR-328 has strong arrhythmogenic potential through a profound reduction of *CACNA1C* and *CACNB1* and shortening of atrial action potential duration (APD) which augments the AF vulnerability [147]. As it has been previously mentioned, miR-499 is increased in AF patients and apart from regulating *SK3* expression, it is also able to directly target *CACNB2* [148] (Table 3). Meanwhile, miR-106b-25 cluster deficiency leads to atrial arrhythmogenesis via enhanced RyR2-mediated SR Ca^2+^-release [149] and miR-208b also reduces the expression and function of the sarcoplasmic reticulum-Ca^2+^ pump *SERCA2* [146] (Table 3).

At the same time, it has been elucidated that the mRNA and protein expression levels of *HCN2* and *HCN4* channels increased with age, whereas miR-1 and miR-133 declined with age, implicating elevated *HCN* activity and reduced miR-1/133-mediated regulation of *HCN* expression in the pathogenesis of AF [150] (Table 3). 

Besides ion channel function, to asses a proper electrical propagation between cardiomyocytes, it is necessary a correct regulation of connexin expression. In this context, miR-206 and miR-208 regulate gap junction protein alpha 1 (*GJA1*) and *GJA5* respectively (Table 3). These miRNAs are increased in AF patients inducing cardiac arrhythmias [151,152], supporting the functional roles of these microRNAs in AF.

Several labs, including ours, demonstrated that Pitx2 deficiency disrupt microRNA expression that are linked to atrial arrhythmogenesis, a signaling path- way that also involves regulation of Wnt and Wnt- driven microRNAs expression, which is highly susceptible to alteration of cardiovascular risk factors such as hyperthyroidism, hypertension and redox homeostasis imbalance [130,153,154,155]. In sum, all these data support the notion that microRNAs play a fundamental role regulating key components that, if impaired, lead to AF electrical remodeling.

#### 4.1.2. microRNAs and Structural Remodeling in AF

Structural remodeling is a long-lasting process that progressively affects myocytes and the myocardial interstitium, and takes place as early as the first days of atrial tachyarrhythmia onset [156]. miRNAs are involved in this process through gene regulation of proteins related to extracellular matrix deposition, apoptosis, and contractility.

Several miRNAs have been identified as potential participants in the regulation of the fibrotic remodeling occurring during AF. miR-21 represses sprouty RTK signaling antagonist 1 (*SPRY1*), a negative regulator of the extracellular signal-regulated kinase (*ERK*) pathway. In AF, *ERK* pathway is activated and promotes fibrosis indirectly through miR-21-induced *SPRY1* downregulation [157]. Additionality, miR-21 also promotes cardiac fibrosis through the transcription factor signal transducer and activator of transcription 3 (*STAT3*) signaling pathway, by decreasing the expression of cell adhesion molecule 1 (*CADM1*) [158]. Finally, at the same time that miR-21 is up-regulated, WW domain containing E3 ubiquitin protein ligase 1 (*WWP1*) expression levels are down-regulated, promoting the activation of TGF-β1/Smad2 signaling pathway which endorses cardiac fibroblasts proliferation in AF patients [159]. By other hand, miR-23b and miR-27b overexpression enhance up-regulation of fibrosis-associated genes by targeting transforming growth factor beta receptor 3 (*TGFBR3*) [160] and posterior activation of SMAD3 signaling. Furthermore, miR-26 modulates Ca^2+^-permeable transient receptor potential canonical-3 (*TRPC3*) protein. miR-26 is down-regulated in AF, thus increasing *TRPC3* expression which in turn stimulates fibroblast proliferation, differentiation, and activation [161]. miR-29 targets multiple extracellular matrix genes, including collagens, fibrillins and elastin, this miRNA is downregulated and its expression is inversely correlated with extracellular matrix protein levels and the development of AF [162]. In this context, miR-30a up-regulation reduces AF-induced myocardial fibrosis by targeting snail family transcriptional repressor 1 (*SNAIL1*) [163], whereas, miR-30c overexpression attenuates atrial fibrosis induced by *TGF-β1*, by targeting transforming growth factor beta receptor 2 (*TGFβRII*) [164], being both of them down-regulated in AF patients with an increase of fibrotic tissue. In addition, it has been demonstrated that miR-30, miR-133 and miR-132 regulate connective tissue growth factor (*CTGF*), which is a key molecule in the process of fibrosis, and collagen production, these miRNAs are down-regulated in AF patients promoting thus atrial fibrosis [165,166]. Also, it has been detected, that nicotine promotes AF by inducing atrial structural remodeling, through miR-133 and miR-590 downregulation and de-repression of *TGF-β1* and *TGFβRII* [167]. Moreover, miR-146b-5p, matrix metallopeptidase 9 (*MMP-9*), involved in the degradation of extracellular matrix and formation of fibrosis, and collagen content were upregulated whereas tissue inhibitor of metalloproteinase 4 (*TIMP-4*) was downregulated in patients with AF [168]. Finally, AF patients showed a drastically increase of myosin heavy chain 7 (*MYH7*) protein levels, a hallmark of cardiac hypertrophy. It is suggested that the increased expression of miR-208a/b in AF contributes to high *MYH7* protein levels via inhibiting the expression of SRY-box transcription factor 5 (*SOX5*) and *SOX6,* however the mechanistic implications of *MYH7* in AF remain unclear [146].

Another layer of regulation of anatomical/structural components by miRNAs is apoptotic cell death, it has been demonstrated that miR-122 is up-regulated in AF patients, inhibiting ERK activation that leads apoptosis. In contrast, miR-133 has a cardioprotective role dependent on *AKT* serine/threonine kinase (*AKT*) signaling in control situation, inducing apoptosis in AF patients due to its down-regulation [169,170].

Apart from electrical and structural remodeling associated to AF, other miRNAs are involved in AF targeting related pathways, i.e., miR-21 modulates Phosphatase and Tensin Homolog (*PTEN*)/Phosphoinositide 3-kinase (*PI3K*) signaling pathway, signal transducer of transcription 3 (*STAT3*) and Smad7 promoting atrial fibrosis; miR-31 begets arrhythmia by depleting dystrophin and neuronal nitric oxide synthase (*nNOS*); miR-34a is upregulated in AF patients having an important role in the early electrophysiological changes and development of AF via regulation of the expression of Ankyrin-B (*ANK 2*); and finally, miR-199a down-regulation induces Sirtuin 1 (*SIRT-1*), a cardio-protective protein, as a compensatory mechanism to inhibit the process of oxidative stress which contributes to the pathogenesis of AF [171,172,173,174,175,176].

All these data support the role of miRNAs in AF pathophysiology. Functional studies targeting miRNAs are necessary to study the therapeutic potential of these molecules in treating cardiovascular disease, although there are multiple concerns as to the safety of miRNA therapeutics, as miRNAs’ ability to target multiple pathways within the target tissue or in different organs, with further research being needed to confirm the safety of miRNAs.

### 4.2. lncRNAs and Atrial Fibrillation

Long non-coding RNAs (lncRNAs) are currently defined as noncoding RNAs large that 200 nucleotides. LncRNAs constitute a widely diverse group of non-coding RNAs with structural similarities to protein-coding RNAs but with no or limited capacity to code for proteins. LncRNAs display a variety of transcriptional and post-transcriptional functions, such as scaffold platform, modulation of epigenetic factors and protein translation among others. Our current understanding of the expression and functional role of long noncoding RNAs (lncRNAs) in human AF is still incipient. Several transcriptomics analyses have been performed, identifying a large array of differentially expressed lncRNAs [177,178,179,180,181,182] in AF. Some of these studies were performed in lone AF patients [179,180,181], whereas in others valvular heart disease [177] or rheumatic valve disease [178,180,182] was also concurring. In most cases, either right or left atrial appendages were analysed [175,176,177,178,179,180], but in some cases right and left atrial samples were pooled together [179,180,181], while in other blood samples [180,183] or epicardial adipose tissue [184,185] were analysed. Given the wide variability of biological conditions studied, it is then not surprising that comparative analyses of these lncRNA transcriptomic analyses revealed no common AF signature [186]. Importantly, concurrent analyses of differentially expressed mRNAs and/or microRNAs and more recently circular RNAs (circRNAs) have provided additional insights into the plausible gene regulatory networks involved in AF [177,178,184,187]. Unfortunately, to date, only the lncRNA fingerprints have been provided and functional assays are scarcely reported. 

At present, assays of the functional role of lncRNAs have only been reported in experimental animal models or in in vitro assays. Several of these studied reported a functional role of lncRNAs modulating fibrosis [188,189,190,191], ion channel function [192,193,194,195,196,197], and energy metabolism [198] as detailed below. 

#### 4.2.1. lcnRNAs in AF Structural Remodelling

Four distinct studies have reported the functional role of lncRNA in AF fibrosis. Cao et al. [189] reported increased PVT1 lncRNA expression in human AF atrial biopsies and furthermore they demonstrate a role for PVT1 enhancing atrial fibroblast proliferation and collagen deposition by sponging miR-128-3p that in turn promoted Tgfb/Smads signaling. Additionally, indirect reports of the functional role of lncRNA in atrial fibrosis have been reported by Lu et al. [190] and Chen et al. [191]. Lu et al. [190] reported significantly reduced GAS5 expression in the right atrial appendage (RAA) of AF patients while GAS5 manipulation in AC16 cells, controlled cell growth by modulating ALK5 expression. Chen et al. [191] demonstrated increased PCAT-1 expression in right atrial appendage of AF patients. Knockdown of PCAT-1 inhibited proliferation in AC16 cell by modulating transforming growth factor-β1 (TGF-β1). Finally, Sun et al. [193] demonstrated that NRON overexpression suppressed, while silencing facilitated, angiotensin II (Ang II)-induced inflammatory response in primary cultured atrial myocytes. Chromatin immunoprecipitation (ChIP) assays showed that nuclear factor of activated T cell 3 (NFATc3) was recruited to the promoter region of interleukin 12 activating its expression in atrial myocytes. Collectively, the authors demonstrated that lncRNA NRON alleviates atrial fibrosis through suppression of M1 macrophages activated by atrial myocytes.

#### 4.2.2. lcnRNAs in AF Electrical Remodelling

Several reports provided evidence on the functional role of lncRNAs modulating the cardiac electrophysiological properties in AF, particularly those related to calcium regulation and handling. Shen et al. [194] reported that KCNQ1OT1 is up-regulated in AngII-treated mice as well as in an experimental AF mouse model. The authors demonstrate that KCNQ1OT1 regulates CACNA1C by sponging miR-384. KCNQ1OT1 manipulation modulate distinct electrophysiological parameters such as the effective refractory period and the interatrial conduction and KCNQ1OT1 silencing diminishes the incidence of AF and AF episodes in AngII-treated mice (Table 4). 

Li et al. [192] reported the lncRNA expression profiles of right atria in AF and non-AF rabbit models and identified 1220 differentially expressed transcripts. TCONS_00075467 was selected for further exploration. In vivo silencing of TCONS_00075467 leading to shortening of the atrial effective refractory period and the L-type calcium current and action potential duration were decreased in vitro. Additionally, the authors demonstrated that TCONS_00075467 sponge miRNA-328 both in vitro and in vivo thus regulating CACNA1C. (Table 4).

More recently, it has been reported that AF patients displayed increased miR-24 and reduced LINC00472 expression, while LINC00472 DNA promoter methylation was also increased. Functional evidence demonstrated that miR-24 can negatively regulate LINC00472 and JP2 expression, and thus LINC00472 could regulate the progression of AF via modulating the LINC00472/miR-24/JP2/RyR2 signaling pathway [195] (Table 4).

The modulative effects of lncRNAs on autonomic neural function and myocardial functions in atrial fibrillation rat model have been also recently investigated [196]. These authors show that over-expression of TCONS_00202959 in an experimental rat AF model enhances the atrial effective refractory period and diminishes the AF induction rate. However, the precise molecular mechanisms by which AERP is decreased remains unclear (Table 4). Similarly, Wang et al. [197] reported the fat pads lncRNA profile in an AF experimental canine model. These authors reported 166 down-regulated and 410 up-regulated (576 differentially expressed lncRNA) lncRNAs and they further underwent to dissect the functional role of two of these differentially expressed lncRNAs, TCONS_00032546 and TCONS_00026102, by in vivo silencing, leading to a significant shortening or prolongation the atrial effective refractory period, and thereby these lncRNAs increased or prevented AF inducibility, respectively (Table 4).

More recently it has been reported complementary expression patterns for MIAT and miR-133a-3p in an experimental AF rat model as well as in peripheral blood leukocyte samples of AF patients [198]. These authors further demonstrated that miR-133a-3p directly regulates by MIAT. MIAT knockdown significantly reverted AF, increasing atrial effective refractory period and thus reducing the duration of AF. Importantly, cardiomyocyte apoptosis and atrial fibrosis were also reduced (Table 4). 

A functional role for lncRNAs in cardiomyocyte metabolism was reported by Chen et al. [199]. These authors performed a microarray analysis using pulmonary vein myocardium and the surrounding myocardium and compared to LA appendage leading to the identification of 94 differentially expressed lncRNAs, among which AK055347 was one of lncRNAs most significantly altered. Experimental manipulation of this lncRNA further demonstrate a role in mitochondrial energy production. In sum, these data support an emerging functional role for lncRNAs in AF.

### 4.3. DNA Methylation and Atrial Fibrillation

DNA methylation is a pre-transcriptional modification, which is able to change transcriptional process, by the addition of methyl groups to specific nucleotides of the DNA. This procedure causes inactive gene expression due to the fact that the methyl binding protein prevents the transcriptional factor from binding to DNA and thus it proceed to the next step [200,201,202]. It is commonly believed that hypomethylation in diseases is more frequent than hypermethylation [203]. 

However, in AF context, global DNA methylation levels are significantly increased in AF patients, having a positive correlation with age [202]. Furthermore, it has been demonstrated that DNA methylation plays an important role in the maintenance of cardiac fibrosis, where DNA methyltransferases 3A (*DNMT3A*) likely plays an essential role in Ras association domain family member 1A (*RASSF1A*) mediated up-regulation of *ERK1/2* [204,205]. Moreover, heart failure induces Pitx2c promoter hypermethylation and Angiotensin II may contribute to the hypermethylation in heart failure [206]. In addition, tumor necrosis factor- α (*TNF-α*) decreases SERCA2 expression via DNMT1 which induces promoter methylation in cardiomyocytes [207]. Emelia’s lab has identified two CpG sites significantly associated with prevalent AF, and five CpGs associated with incident AF, and fourteen previously reported genome-wide significant AF-related SNP were each associated with at least one CpG site; being the most significant association rs6490029 at the CUX2 locus and cg10833066 [208]. Recently studies have shown that KIF15 methylation may play important role in the pathogenesis of AF through the regulation of the expression of proteasome 26S subunit ATPase 3 (*PSMC3*), tubulointerstitial nephritis antigen (*TINAG*), and nudix hydrolase 6 (*NUDT6*) [209].

These results suggest that DNA methylation might represent an important molecular process that is able to link genetic variations with AF susceptibility. To date, only a few studies have investigated differential DNA methylation as a predictor biomarker at specific candidate loci that were previously associated with AF. 

### 4.4. Histone Modifications and HDACs in AF

Histone modification represent an important mechanism of epigenetic regulation. The N-terminal of histones can undergo distinct post-transcriptional modifications and the most common modifications include phosphorylation, acetylation, methylation, and ubiquitination, but others occur as well [210]. Such post-transcriptional modifications thus play important biological roles in a wide array of cellular processes including cell cycle and metabolism control, DNA repair and particularly important on gene transcription. To date, only post-transcriptional modification by acetylation has been reported in association to AF.

Histone acetylation, modulated by histone acetyltransferases (HATs) is normally associated to open chromatin configurations and thus to active gene transcription while histone deacetylation, catalysed by distinct classes of histone deacetylases (HDACs) is linked to gene silencing [201]. Currently, our understanding of the functional impact of HDAC in histone modification in the setting of AF remains largely unexplored. However, HDAC, besides post-transcriptionally regulating histones and the nuclear chromatin, can also be translocated into the cytoplasm modulating acetylation and deacetylation of other proteins [211,212,213]. In this context, emerging evidence is demonstrating a pivotal role of HDACs influencing post-transcriptional regulation of distinct proteins in cardiomyocytes in the context of AF [214,215], particularly on cytoskeletal [213] and conductive proteins [216], while their role in contractile and ion channels remains unclear [217]. Additionally, HDAC inhibition can significantly block or halt AF progression [216,218,219,220,221], further supporting the important role of HDAC in AF, yet the molecular mechanisms remain to be further explored.

## 5. Therapeutic Consequences of Ion-Channel Remodelling

Ion-channel remodelling represents a potential antiarrhythmic drug target. There are some channel blockers which are more effective than others, particularly those that modulate T-type Ca^2+^-channels, having a superior efficacy, over those that act on I_Ca,L_, K^+^-channel and Na^+^-channel which are mostly ineffective. For example, Mibefradil and Amiodarone are T-type Ca^2+^-channel blockers that suppress APD shortening [222] while Bepridil, a L- and T-type Ca^2+^-channel blocker, suppresses ion-channel remodelling, promotes long ERP, low AF inducibility and AF duration is shorter [223]. There are some other drugs that work at atrial but not at ventricular levels [224] such as for example, AVE0118 that acts over I_kur_ in atrial appendages, reducing the APD in chronic AF [225], and Tertiapin an I_K,ACh_-blocker, that prolongs APD in ATR-remodelled canine preparations and thus suppresses tachyarrhythmias [226]. Concomitant administration of Flecainide and AVE0118 have the ability to inhibit constitutive I_K,ACh_ in chronic AF patients, an effect that might contribute to their effectiveness in terminating AF [227].Therefore, therapeutics targeting ion channels could be useful in an early cardioversion strategy.

## 6. Perspectives

Atrial fibrillation is the most common cardiac arrhythmia in the general population and thus great efforts are continuously done to understanding the molecular substrates underlying AF. We have witnessed over the last decades a great advance on the discovery of the genetic bases of AF. First genetic analyses were mostly conducted as candidate approaches taking as substrates those ion channels configuring the cardiac action potential and following the reasoning of guilty by association. A large number of mutations were revealed in this way. However, functional assays were compulsory to distinguish the needle on the haystack and the results were in several cases hard to reconcile. In addition, the pathophysiology of AF was progressively deciphered demonstrating that, besides electrophysiological disturbances, extracellular matrix deposition, inflammation, and metabolic disorders also contributed to the onset and progression of AF, thus broadening the spectrum of candidate approaches. The advent of novel genetic approaches, such as genome wide association analyses (GWAS), circumvented in part those limitations. GWAS analyses have revealed over 100 risk variants associated with AF [12]. In most cases, those AF associated variants are located in intergenic regions, limiting our understanding of the molecular mechanisms behind those associations. The exception to the rule is represented by 4q25 variants, to which several studies, including ours, have demonstrated a pivotal role regulating Pitx2 expression [228,229,230] as well as demonstrating a functional role for PITX2 in AF pathophysiology [130,133,134,154]. Thus, one of the future challenges in the genetics of AF is to discover the molecular mechanism behind GWAS data and AF pathophysiology [231], reemphasizing the responsibility of epigenetics. 

Epigenetic regulation of AF is being progressively deciphered. DNA methylation and histone modifications implication in AF is still in its infancy whereas the functional role of microRNAs and lncRNAs have been already dissected in distinct AF pathophysiological settings, including their role on ion channel regulation, extracellular matrix deposition and fibrosis, inflammation, and metabolism. In coming years, we will therefore witness an explosion of studies unravelling the contribution of epigenetic mechanisms to AF associated substrates such as gene regulatory networks linking DNA methylation and/or histone modifications to transcriptional regulation of key AF-associated transcription factors such as PITX2, TBX5, and ZFHX3 among others, or complex lncRNA–microRNA–mRNAs gene regulatory networks impacting on the electrophysiological and structural remodelling substrates underlying AF.

## Figures and Tables

**Table 1:** Summary of the genes associated with atrial fibrillation (AF).

Genes	Function	Type of Mutations	Functional Effect in the AF Context
**Potassium channels**			
*ABCC9*	ATP-binding cassette, subfamily C, member 9	LoF	Uncertain
*KCNA5*	α-Subunit of voltage-gated potassium channel Kv1.5	GoF; LoF	Promote AF initiation
*KCND3*	α-Subunit of voltage-gated potassium channel Kv4.3	GoF	Enhance AF susceptibility
*KCNE1*	β-Subunit of voltage-gated potassium channel Kv7.1	GoF	Increase I_Ks_ and exert potential effect on I_to_ and I_Kr_
*KCNE2*	β-Subunit of voltage-gated potassium channel Kv7.2	GoF	Increase I_Ks_ and exert potential effect on I_to_ and I_Kr_
*KCNE3*	β-Subunit of voltage-gated potassium channel Kv7.3	GoF	Increase I_Ks_ and exert potential effect on I_to_ and I_Kr_
*KCNE4*	β-Subunit of voltage-gated potassium channel Kv7.4	Uncertain	Increase I_Ks_ and exert potential effect on I_to_ and I_Kr_
*KCNE5*	β-Subunit of voltage-gated potassium channel Kv7.5	GoF	Increase I_Ks_ and exert potential effect on I_to_ and I_Kr_
*KCNH2*	α-Subunit of voltage-gated potassium channel Kv11.1	GoF; LoF	Induce frequent paroxysms of AF
*KCNJ2*	α-Subunit of inwardly rectifying potassium channel Kir2.1	GoF	Promote AF initiation
*KCNJ5*	α-Subunit of inwardly rectifying potassium channel Kir3.4	GoF	Promote AF initiation
*KCNJ8*	α-Subunit of inwardly rectifying potassium channel Kir6.1	NA	Promote AF initiation
*KCNN3*	Intermediate/small conductance calcium-activated potassium channel, KCa2.3	GoF	Uncertain
*KCNQ1*	α-subunit of voltage-gated potassium channel Kv7.1	GoF; LoF	Induce altered I_Ks_ activity and kinetics, increase the arrhythmogenicity to AF, most likely by shortening atrial but not ventricular action potential
***Sodium/potassium channels***			
*HCN4*	Hyperpolarization activated cyclic nucleotide-gated potassium channel 4	LoF	May lead to diminished action potential frequency (heart rate slowing) and delayed after depolarizations that might trigger AF
***Sodium channels***			
*SCN1B*	β-Subunit of Nav1.5, type I	LoF	Attenuate sodium currents and shift the voltage-dependence of sodium channel gating and is associated to AF
*SCN2B*	β-Subunit of Nav1.5, type II	LoF	Attenuate sodium currents and shift the voltage-dependence of sodium channel gating and is associated to AF
*SCN3B*	β-Subunit of Nav1.5, type III	LoF	Attenuate sodium currents and shift the voltage-dependence of sodium channel gating and is associated to AF
*SCN4B*	β-Subunit of Nav1.5, type IV	LoF	Attenuate sodium currents and shift the voltage-dependence of sodium channel gating and is associated to AF
*SCN5A*	α-Subunit of Nav1.5	GoF; LoF	Constitute a substrate of early-onset AF
*SCN10A*	α-Subunit of Nav1.8	GoF; LoF	Promote early onset of AF
***Genes involved in calcium homeostasis***			
*RyR2*	Ryanodine receptor 2	GoF	Modulate AF susceptibility by altering the calcium homeostasis
*CACNB2*	β2-subunit of the L-type calcium channel	Uncertain	Modulate AF susceptibility by altering the calcium homeostasis
*CACNA2D4*	l-type calcium-channel auxiliary subunit of the alpha-2/delta subunit family	Uncertain	Modulate AF susceptibility by altering the calcium homeostasis
***Genes involved in fibrosis and extracellular matrix (ECM) remodeling***			
*NPPA*	Natriuretic peptide precursor A	GoF	May promote to AF by activating inflammation and fibrosis
*MMP3*	Member of the matrix metalloprotease family	Uncertain	May promote atrial fibrosis and trigger structural remodeling in the atria
*COMP*	Cartilage oligomeric matrix protein	Uncertain	May promote atrial fibrosis and trigger structural remodeling in the atria
*COL12A1*	Collagen alpha-1(XII) chain	Uncertain	May promote atrial fibrosis and trigger structural remodeling in the atria
*COL23A1*	Collagen α-1 (XXIII) chain	Uncertain	May promote atrial fibrosis and trigger structural remodeling in the atria
*COL21A1*	Collagen alpha-1(XXI) chain	Uncertain	May promote atrial fibrosis and trigger structural remodeling in the atria
*ANGPTL2*	Angiopoietin-like protein 2	Uncertain	May promote atrial fibrosis and trigger structural remodeling in the atria
*COLQ*	Acetylcholinesterase-associated collagen	Uncertain	May promote atrial fibrosis and trigger structural remodeling in the atria
***Genes involved in cardiogenesis***			
*GATA4*	GATA Binding Protein 4	LoF	Decreased transcriptional activity
*GATA5*	GATA Binding Protein 5	LoF	Decreased transcriptional activity
*GATA6*	GATA Binding Protein 6	LoF	Decreased transcriptional activity
*GREM2*	Gremlin-2	GoF	Involvement in the early onset of AF
*NKX2-5*	Homeobox protein Nkx-2.5, transcription factor	Uncertain	Causally implicated in AF
*NKX2-6*	Homeobox protein Nkx-2.6, transcription factor	Uncertain	Causally implicated in AF
***Genes implicated in the cell-cell coupling***			
*GJA1*	Connexin 43	GoF; LoF	Impaired trafficking or channel formation
*GJA5*	Connexin 40	GoF; LoF	Impaired trafficking or channel formation
***Genes implicated in nuclear structure***			
*LMNA*	Lamin A/C	Uncertain	Impaired interaction between lamin A/C and NUP155
*NUP155*	Nucleoporin	LoF	Impaired interaction between lamin A/C and NUP156

GoF: Gain-of-function; LoF: Loss-of-function.

**Table 2:** Main genetic loci identified by GWAS in association with AF.

SNP ID	Locus	Nearest Gene	Position Relative to the Gene
rs6666258	1q21	*KCNN3*	Intronic
rs13376333	1q21	*KCNN3*	Intronic
rs3903239	1q24	*PRRX1*	Intergenic
rs4642101	3q25	*CAND2*	Intronic
rs6817105	4q25	*PITX2*	Intergenic
rs2200733	4q25	*PITX2*	Intergenic
rs2040862	5q31	*WNT8A*	Intronic
rs13216675	6q22	*GJA1*	Intergenic
rs3807989	7q31	*CAV1/2*	Intronic
rs10821415	9q22	*C9ORF3*	Intronic
rs10824026	10q22	*SYNPO2L/MYOZ1*	Intergenic
rs12415501	10q24	*NEURL*	Intronic
rs6584555	10q24	*NEURL*	Intronic
rs10507248	12q24	*TBX5*	Intronic
rs6490029	12q24	*CUX2*	Intronic
rs1152591	14q23	*SYNE2*	Intronic
rs7164883	15q24	*HCN4*	Intronic
rs2106261	16q22	*ZFHX3*	Intronic

**Table 3:** AF associated microRNAs and their functional consequences in AF electrophysiology.

Gene	Targets	Regulatory Role	AF Related Functional Consequences	Reference
*miR-1*	*KCNJ2*	increased IK1 current	increased membrane resting potential; increased AF vulnerability	[138]
	*KCNE1*	increased Iks current	decreased AERP; increased AF vulnerability	[140]
	*KCNB2*	increased Iks current	decreased AERP; increased AF vulnerability	[140]
	*HCN2*	increased expression	plausible increase in premature beats; increased AF vulnerability	[150]
	*HCN4*	increased expression	plausible increase in premature beats; increased AF vulnerability	[150]
*miR-26*	*KCNJ2*	increased IK1 current	increased membrane resting potential; increased AF vulnerability	[139]
*miR-30d*	*KCNJ3*	reduced IK.Ach current	impaired calcium handling; increased AF vulnerability	[141]
*miR-499*	*KCNN3*	reduced SK3 expression	no direct evidences to AF pathophysiology	[142]
*miR-192*	*SCN5A*	reduced Nav1.5 expression	no direct evidences to AF pathophysiology	[143]
*miR-21*	*CACNA1C*	reduced Ica current	shortening APD; increased AF vulneratibility	[144]
	*CACNB2*	reduced Ica current	shortening APD; increased AF vulneratibility	[144]
*miR-29*	*CACNA1C*	reduced Ica current	no direct evidences to AF pathophysiology	[145]
*miR-208ab*	*CACNA1C*	reduced expression	potential impact in APD and thus on AF vulneratibility	[148]
	*CACNB2*	reduced expression	potential impact in APD and thus on AF vulneratibility	[148]
	*GJA5*	indirect reduced expression	no direct evidences to AF pathophysiology	[152]
	*ATP2A2*	reduced expression	no direct evidences to AF pathophysiology	[146]
*miR-328*	*CACNA1C*	reduced Ica current	shortening APD; increased AF vulneratibility	[147]
	*CACNB2*	reduced Ica current	shortening APD; increased AF vulneratibility	[147]
*miR-106b-25*	*RYR2*	increased Ca++ release	increased pacing-induced AF vulnerability	[149]
*miR-206*	*GJA1*	reduced Cx43 expression	abnormal heart rate and PR interval; plausible link to AF	[151]

**Table 4:** AF associated lncRNAs and their functional consequences in AF electrophysiology.

Gene	Targets	Regulatory Role	AF Related Functional Consequences	Reference
KCNQ1OT1	*CACNA1C*	miR-384 sponge	impaired AERP and the interatrial conduction; increased AF vunerability	[194]
TCONS_00075467	*CACNA1C*	miR-328 sponge	reduced ICa and shortened APD and AERP; increased AF vunerability	[192]
LINC00472	unknown	miR24/JP2/RyR2	no direct evidences to AF pathophysiology	[195]
TCONS_00202959	unknown	unknown	shortened AERP and increased AF vunerability	[196]
TCONS_00032546	unknown	unknown	shortened AERP and increased AF vunerability	[197]
TCONS_00026102	unknown	unknown	increased AERP and prevented AF inducibility	[197]
MIAT	unknown	miR-133 sponge	increased AERP and prevented AF inducibility	[198]

## Abbreviations

AF	Atrial fibrillation
GWAS	Genome-wide association studies
CVDs	Cardiovascular diseases
ECG	Electrocardiogram
AV	Atrioventricular
SR	Sarcoplasmic reticulum
RyRs	Ryanodine receptors
ECM	Extracellular matrix
LQT3	Long QT syndrome type 3
BrS	Brugada Syndrome
WES	Whole Exome Study
TF	Transcription factors
GJs	Gap junction
SNP	Single nucleotide polymorphisms
miRNAs	MicroRNAs
AERP	Effective refractory period
APD	Atrial action potential duration
LncRNAs	Long noncoding RNAs
CircRNAs	Circular RNAs
